# PIIKA 2: An Expanded, Web-Based Platform for Analysis of Kinome Microarray Data

**DOI:** 10.1371/journal.pone.0080837

**Published:** 2013-11-29

**Authors:** Brett Trost, Jason Kindrachuk, Pekka Määttänen, Scott Napper, Anthony Kusalik

**Affiliations:** 1 Department of Computer Science, University of Saskatchewan, Saskatoon, Saskatchewan, Canada; 2 Emerging Viral Pathogens Section, National Institute of Allergy and Infectious Diseases, National Institutes of Health, Frederick, Maryland, United States of America; 3 Vaccine and Infectious Disease Organization, University of Saskatchewan, Saskatoon, Saskatchewan, Canada; 4 Department of Biochemistry, University of Saskatchewan, Saskatoon, Saskatchewan, Canada; Huazhong University of Science and Technology, China

## Abstract

Kinome microarrays are comprised of peptides that act as phosphorylation targets for protein kinases. This platform is growing in popularity due to its ability to measure phosphorylation-mediated cellular signaling in a high-throughput manner. While software for analyzing data from DNA microarrays has also been used for kinome arrays, differences between the two technologies and associated biologies previously led us to develop Platform for Intelligent, Integrated Kinome Analysis (PIIKA), a software tool customized for the analysis of data from kinome arrays. Here, we report the development of PIIKA 2, a significantly improved version with new features and improvements in the areas of clustering, statistical analysis, and data visualization. Among other additions to the original PIIKA, PIIKA 2 now allows the user to: evaluate statistically how well groups of samples cluster together; identify sets of peptides that have consistent phosphorylation patterns among groups of samples; perform hierarchical clustering analysis with bootstrapping; view false negative probabilities and positive and negative predictive values for t-tests between pairs of samples; easily assess experimental reproducibility; and visualize the data using volcano plots, scatterplots, and interactive three-dimensional principal component analyses. Also new in PIIKA 2 is a web-based interface, which allows users unfamiliar with command-line tools to easily provide input and download the results. Collectively, the additions and improvements described here enhance both the breadth and depth of analyses available, simplify the user interface, and make the software an even more valuable tool for the analysis of kinome microarray data. Both the web-based and stand-alone versions of PIIKA 2 can be accessed via http://saphire.usask.ca.

## Introduction

Catalyzed by protein kinases, reversible protein phosphorylation is the most widespread signaling mechanism in eukaryotes and plays a critical role in virtually every cellular process. Technologies for studying phosphorylation-mediated signaling in a high-throughput manner have the potential to facilitate the discovery of complex biomarkers, help identify signaling pathways associated with particular diseases, and provide general information regarding regulatory mechanisms. One such technology is the kinome microarray, in which natural substrates of protein kinases are mimicked by short (15-mer) peptides containing the phosphoacceptor site (at the central position) as well as the same surrounding residues as in the corresponding intact protein. The phosphorylation kinetics of these peptides and their corresponding proteins are similar [1], [2]. First proposed in 2002 [3], [4], kinome arrays have since been used to study a large variety of biological systems, such as the effects of glucocorticoids on the immune system [5], signaling in chondrosarcoma [6], sugar signaling in plants [7], [8], stem cell differentiation [9], bacterial infections in cows [10], [11], and many others [12].

Previously, researchers using kinome microarrays have analyzed the resulting data using software designed for DNA microarrays. However, the chemistry involved in the two technologies is different, and data processing appropriate for one technology may not be appropriate for the other. Further, given the smaller number of spots on a kinome array (∼300–1000) versus a DNA array (∼30,000), the use of the same statistical stringency thresholds commonly employed in DNA array software could compromise the ability to identify differentially phosphorylated peptides in kinome arrays and to identify changes in the modulation of biological pathways. DNA microarray software also often lacks statistical techniques for ascertaining the consistency of technical and biological replicates. In response to these concerns, we developed a software program in the R environment [13] called Platform for Intelligent, Integrated Kinome Analysis (PIIKA) [14], and showed that it improves the ability to identify cellular signaling pathways that are upregulated or downregulated in response to a particular treatment. PIIKA also facilitates the identification of peptides that have inconsistent responses among the technical replicates on a single array or among different biological replicates (e.g. different animals exposed to the same treatment), ensuring that only high-quality data are used in subsequent statistical and clustering analyses.

Here, we report the development and release of PIIKA 2, which contains many additions and improvements to PIIKA, primarily in the categories of cluster analysis, statistical analysis, and data visualization. Among others, PIIKA 2 allows users to perform the following tasks, which would have been impossible in the original PIIKA without substantial user effort (e.g. writing of scripts):

- determine the statistical significance of the consistency between the actual clustering of the data and a hypothesized clustering;
- identify subsets of peptides that induce a particular clustering;
- assess the statistical significance of hierarchical clustering nodes using bootstrapping analysis;
- quickly access false negative rates and positive and negative predictive values for the t-tests between pairs of samples;
- easily evaluate the technical and biological reproducibility of the experiment;
- visualize principal component analysis (PCA) results using a three-dimensional interactive plot;
- visualize points that are both statistically significant and have high fold-change values using volcano plots; and
- view the relationships between the normalized signal intensities in pairs of samples.

In summary, PIIKA 2 improves the ability to answer complex biological questions about kinome array data and to make informed decisions concerning statistical thresholds and significance. Whereas the original PIIKA was available only as a command-line tool, PIIKA 2 may also be used via a web-based interface, which eases the data analysis process for users unfamiliar with the use of command line tools. A significant advantage of PIIKA 2 over stand-alone graphical user interface (GUI)-based tools is that there is no need to click on menu items and change options for each individual analysis the user would like to perform. PIIKA 2 performs all analyses that are applicable given the input provided by the user and outputs the results in the form of spreadsheet-compatible text files and publication-ready images.

As mentioned, PIIKA 2 is available in two forms: a web-based version, and a local version that can be installed on the user's computer. Both versions are available through the Saskatchewan PHosphorylation Internet REsource (SAPHIRE) website at http://saphire.usask.ca. PIIKA 2 is free for academic use; users interested in PIIKA 2 for commercial purposes should contact the authors.

The remainder of this paper is divided into three major sections. The Methods section discusses the methodology associated with each new feature of PIIKA 2. The Results section gives examples and figures that illustrate the application of these features to data from a real kinome microarray experiment. Finally, the Discussion and conclusion section summarizes the value of PIIKA 2 for analyzing kinome array data and discusses the utility of kinome arrays for signaling research in general.

## Methods

When dealing with complex data such as those arising from kinome microarrays, asking non-trivial questions of the data often requires expertise in mathematics, programming and data visualization—as well as a significant investment of time. Ultimately, these often deter users from interrogating their data to the full extent possible. To address this problem, we have implemented in PIIKA 2 a rich assortment of analysis tools. These tools relate to cluster analysis, statistical analysis, or data visualization. As we receive feedback from users, other functionality will be added. This section contains descriptions of the methodologies used; for examples of the use of these methodologies, including relevant figures and example outputs, see the Results section.

### Cluster analysis

The original version of PIIKA allowed users to perform hierarchical clustering on the samples in a given experiment; however, the tools available to analyze the clusters were limited. Here, three features new to PIIKA 2 are described that allow users to perform more detailed analyses of their hierarchical clustering results.

### Random tree analysis: statistical significance of the clustering of *a priori* groups

In many kinome microarray experiments, the samples or treatments can be placed *a priori* in different groups based on either biological knowledge or specific attributes of the samples or treatments. For brevity, in the following discussion the members of these groups will be called “samples”, although if each experimental treatment has more than one sample associated with it, then the members of these groups would more accurately be called “treatments”.

In a real experiment conducted by our research group, for example, one sample was taken from each of 6 biological subjects at each of 4 time points. These samples were then processed using kinome microarrays containing 297 unique peptides, each replicated 9 times on the same array. Image analysis software was used to capture the phosphorylation intensity of each spot as described previously [15], and the resulting data were processed using PIIKA 2. The exact nature of the experiment, the samples, and the subjects is not relevant here (a manuscript describing these data from a biological perspective is in preparation); in this study, the critical feature of the example experiment is that we hypothesize that samples from the same subject will have similar kinome profiles. The original version of PIIKA included functionality for performing hierarchical clustering, which allows the similarity of the kinome profiles of the samples to be ascertained. Although one can get a sense of whether the expected clustering pattern does indeed exist by visually inspecting the resulting dendrogram, this does not give a measure of statistical significance. To remedy this, PIIKA 2 allows the question, “Do samples from the same group cluster together better than would be expected by chance?” to be addressed by deriving an empirical statistical distribution and then reporting a P-value based on this distribution, where a small P-value indicates that samples within the same group (in the above example, the same biological subject) cluster together better than would be expected at random.

Since each step in the process of performing hierarchical clustering results in a bifurcation, clusterings made in this way can always be represented as binary trees. For ease of reference, we therefore convert the dendrogram representation to its corresponding binary tree representation. To evaluate the “goodness” of clustering for a given binary tree 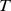, we define a metric 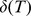 wherein larger values denote better clustering. Suppose that, in our hypothesized grouping of the samples, there are 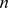 groups labeled 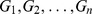, each containing 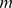 samples. In the example above, 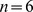 and 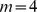. Also, let the internal nodes of 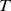 be labeled 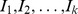, where 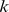 is the number of internal nodes. We define a function 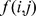 as follows:
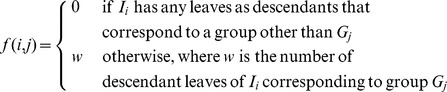



Then
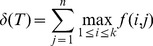
(1)


In other words, to calculate 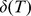, for each group 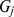 we find the internal node 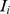 with the greatest number of leaves as descendants that correspond to 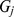 and that has no leaves corresponding to any other group. The number of such leaves is added to 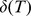. Thus, the maximum possible value of 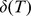 is 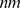, and the possible values of 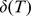 are the integers between 0 and 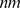. To make the metric independent of 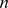 and 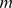, it can be expressed as a ratio: 
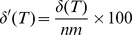
. A 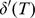 value of 100 indicates perfect clustering. The Results section contains an example of a tree 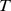 and the calculation of its corresponding score 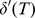.

While 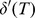 by itself gives a sense of the goodness of clustering, it does not indicate whether the samples from each *a priori* group cluster together better than would be expected at random. To determine this, 10,000 random trees 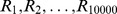 are generated (the number of random trees generated can be changed by the user), and the value of 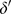 is calculated for each. The random trees are generated by modifying the original data matrix, wherein rows represent peptides and columns represent arrays, by randomly rearranging the values within each column. The values 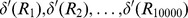 represent an empirical probability distribution for 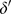. Thus, the P-value is simply the proportion of random trees 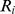 for which 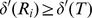. For each 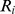, PIIKA 2 outputs the rearranged matrix that was used to produce that random tree, visual and text-based representations of the hierarchical clustering of that matrix, and the value of 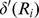. PIIKA 2 also outputs 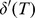 and the aforementioned P-value.

### Peptide subset analysis: identifying sets of peptides that support the clustering of *a priori* groups

Given a set of groups of samples defined *a priori* based on biological knowledge or other factors, it may also be of interest to identify sets of peptides for which the phosphorylation patterns are similar within samples from the same group and different between samples from different groups (as described above, the members of the groups may be either samples or treatments, but for brevity we will just call them “samples”). In other words, one might want to identify sets of peptides for which the clustering of the samples into these groups is as close to perfect as possible. For example, consider a hypothetical experiment in which cell extracts are taken from mice with a genetic propensity to a certain disease, and that we divide these mice into two groups—those that eventually get the disease, and those that do not. If we could identify a set of, say, 10 peptides that have similar responses in mice of the same group, and different responses between groups, then these 10 peptides could potentially act as a biomarker for this disease.

PIIKA 2 implements this functionality using a simple local search procedure. First, the samples (or treatments, if more than one sample corresponds to a particular treatment) are hierarchically clustered using a set of exactly two peptides drawn from the complete set. The score for the corresponding tree (which, again, is a clustering of the samples, not the peptides), 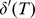, is then determined. This procedure is then repeated for all possible pairs of peptides. The pair of peptides which results in the tree with the greatest value of 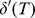 is then selected as the “seed”. If more than one set has the same value of 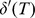, then one of them is arbitrarily chosen to be the seed. A third peptide is then added to this list by scanning the remaining peptides and determining which one—in addition to the two chosen as the seed—results in the set with the greatest value of 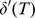. Additional peptides are iteratively added in the same fashion until all peptides have ultimately been added, in which case the dendrogram is identical to the one created using all of the peptides. For each iteration, the hierarchical clustering is performed anew (as opposed to adding the next peptide onto the structure of the previous tree).

PIIKA 2 outputs, for each 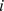 (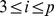, where 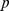 is the number of peptides), the dendrogram containing 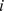 peptides, the score 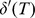 associated with that dendrogram, and a spreadsheet-compatible table showing the names of those peptides as well as their normalized intensity values for each sample. The peptides forming these subsets are those having phosphorylation patterns that are similar within samples from the same group, but different between samples from different groups. Depending on the biological application, it might be of interest to examine small sets of peptides (say, 5 or 10) that have this property, or it might be more meaningful to examine larger sets of peptides. The output of PIIKA 2 allows the user to examine sets of peptides with any cardinality between 3 and the total number of unique peptides.

### Bootstrap analysis of hierarchical clustering

When performing hierarchical clustering, the strength of the support for each cluster can be ascertained using bootstrapping. As a complement to the heatmaps produced by PIIKA, PIIKA 2 also outputs dendrograms showing the hierarchical clustering of the samples, with each node labeled with two P-values: the bootstrap confidence P-value (BP) as proposed by Felenstein [16], and the approximately unbiased P-value (AU) as proposed by Shimodaira [17], [18]. Each P-value ranges between 0 and 100, and represents the percentage of times that the cluster appears in the bootstrap replicates. The R package pvclust [19] is used to calculate these bootstrap values and generate the graphical version of the dendrogram.

It should be noted that the variables (peptides) are not strictly independent, largely because a given kinase might catalyze the phosphorylation of several peptides on the array. This could compromise the statistical soundness of the bootstrap analysis, as each resampling of the original data may not reflect the dependence originally present among the variables. However, similar bootstrap analyses have successfully been used for DNA microarrays (e.g. [20]–[24]), despite the fact that the expression levels of individual genes may not be independent (due, for example, to transcription factors that each promote the transcription of several genes). This suggests that bootstrap analysis should be valuable for kinome arrays as well. Nonetheless, the fact that the peptides are not independent should be kept in mind when interpreting the results.

### Statistical analysis

In the original version of PIIKA, several statistical tests were provided, including a t-test for comparing treatment-control combinations, a 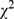-test for identifying peptides inconsistently phosphorylated among the technical replicates, and an F-test for determining the consistency of biological replicates. In this section, we describe statistical analyses performed by PIIKA 2 that were not possible to perform in the original PIIKA.

**False positive and false negative probabilities.** The original version of PIIKA allowed the user to select a value for 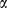 (the probability of a type I error; also called the false positive rate) for the t-tests done between each peptide for a given treatment and control. While controlling the type I error rate is important, it is also important to be cognizant of the type II error rate (denoted 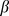, and also called the false negative rate). This is particularly true because subsequent analyses often involving feeding the data into a program like InnateDB [25], which examines whether a particular cellular signaling pathway appears to be upregulated or downregulated based on the increased or decreased phosphorylation of individual components of that pathway. If the false negative rate is too high, then peptides that are differentially phosphorylated may not be correctly identified, causing pathways to be missed that are in fact differentially regulated in the treatment condition compared to the control condition. As such, it could be valuable to the user to display these false negative probabilities.

In its output files that give the t-test results for each peptide for each treatment-control combination, PIIKA 2 now also includes the value of 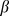 for each peptide. These values are calculated using the R package pwr. Since 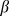 decreases when 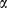 is increased, the user can choose to increase the value of 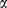 if the values of 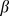 are judged to be too high. Note that increasing the number of intra-array technical replicates will also lower the false negative probabilities, although this is usually not an option at the stage in the experiment where array data have already been gathered.

**Positive and negative predictive values.** Let 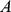 represent the event of rejecting the null hypothesis, and let 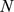 represent the event that the null hypothesis is true. Then the false positive probability 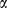 can be defined as 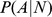. While 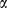 is a useful quantity, sometimes it is more meaningful to know the complementary probability 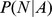 (sometimes called “positive predictive value”)—given that we rejected the null hypothesis, what is the probability that it is true? 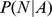 can be calculated mathematically using Bayes' rule: 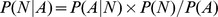. Both 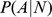 and 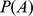 are easy to determine: 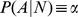, which is supplied by the user, while 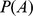 is the proportion of peptides attaining a P-value less than 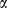. Unfortunately, 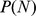 is more difficult to determine, as this represents the actual background probability that a particular peptide will not be differentially phosphorylated. PIIKA 2 uses a (somewhat arbitrary) default of 0.75 for this value, although this can be changed by the user if desired.

Similarly, it may also be useful to find the probability that the null hypothesis is false given that we failed to reject it (sometimes called “negative predictive value”)—that is, 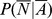. Analogous to the above, this can be determined using Bayes rule: 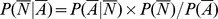. Here, 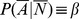, while 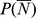 and 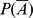 are the complements 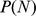 and 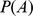, respectively.

As with 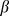, the t-test files produced by PIIKA 2 now include the probabilities 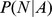 and 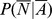 as described above. 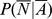 is given as a column in the file, as it potentially will differ for each peptide; however, 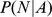 will have the same value for every peptide, so it is listed in a separate file.

**Technical and biological reproducibility summaries.** To facilitate statistical hypothesis testing, kinome arrays typically contain between three and nine intra-array technical replicates; in other words, between three and nine distinct spots are placed on the array for each unique peptide sequence. In the original PIIKA publication [14], we described the use of a 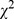-test to identify peptides that are inconsistently phosphorylated among the technical replicates on a single array.

In our own publications describing results from biological experiments involving kinome microarrays (e.g. [11]), we typically include a statement summarizing the technical reproducibility of the phosphorylation signal for all the arrays used in the experiment. For instance, for arrays that each contain 297 unique peptides, we might claim that the average number of consistently phosphorylated peptides on a given array was 288, and that this value ranged from 282 to 296. In the previous version of PIIKA, the user would have had to manually calculate these values from other output. In contrast, PIIKA 2 generates a file containing the number of consistently phosphorylated peptides for all the arrays in the experiment, along with the average value and range of values, making it easy to include this information in a manuscript describing the experiment.

In addition to summarizing technical reproducibility, PIIKA 2 also summarizes the biological reproducibility if the experiment involves more than one biological replicate per treatment. The information presented is analogous to that given in the technical reproducibility summary: for each treatment, the number of peptides consistently phosphorylated among the biological replicates is given, along with the average and range of these values.

### Data visualization

The original version of PIIKA contained three major data visualization methods: heatmaps (showing the hierarchical clustering of samples on the 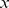 axis and peptides on the 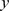 axis), 2-dimensional and 3-dimensional scatterplots showing the results of PCA, and a novel visualization method for comparing differential phosphorylation P-values between two treatment-control combinations [14]. PIIKA 2 provides several additional visualization methods; these are described below.

**PCA visualization using Virtual Reality Modeling Language.** While the first three principal components can be visualized using a 3D scatterplot, as provided in the original PIIKA, it can be difficult to comprehend such plots, especially when there are many samples. The layout of sample labels can also pose problems in 3D scatterplots. As such, interactive plots created using virtual reality modeling language (VRML) are an attractive alternative. PIIKA 2 uses the R package vrmlgen [26]—specifically, the function cloud3d—to generate 3D scatterplots in VRML. Using an appropriate viewer, such as Instant Player (http://www.instantreality.org), the user can rotate and translate the figure, as well as zoom in and out, making the relationship between the samples in three-dimensional space easier to comprehend.

**Volcano plots.** When comparing the level of phosphorylation between a treatment and a control, two quantities are often of interest: the P-value corresponding to the t-test, which answers the question, “Is there a statistically significant difference between the phosphorylation level in the treatment and the phosphorylation level in the control?”, and the fold-change (FC) value, which answers the question, “What is the magnitude of the difference between the phosphorylation level in the treatment compared to the control?”. These quantities are not necessarily meaningful in isolation: very large or very small FC values may be associated with a lot of variability in the technical replicates, and thus have an insignificant P-value according to the t-test; conversely, the magnitude of the difference between the treatment and control may be small, but the technical replicates may be highly consistent within each sample, leading to a small P-value. A useful visualization method for looking at fold-change values and P-values simultaneously is the “volcano plot” [27]— a scatterplot with FC on the 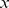-axis and P-value on the 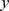-axis, and named as such because the pattern exhibited by the points often resembles an erupting volcano. Points located in the upper-left or upper-right corners of the plot are usually of the most interest, as they have both small P-values and high FC values. PIIKA 2 generates a volcano plot for each treatment-control combination specified by the user.

**Scatterplots between pairs of samples.** In addition to visualizing how different samples are from each other using hierarchical clustering or PCA, it may be useful to compare the normalized intensity values between two samples at a more fine-grained level—i.e. by directly visualizing differences in responses between individual peptides. To facilitate this, PIIKA 2 outputs, for each possible pair of samples, a scatterplot containing a point for each peptide, where a point's 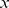 and 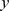 coordinates represent that peptide's normalized intensity value for the first and second sample in the pair, respectively. Each scatterplot also contains a least-squares regression line, the line 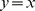 (for comparison to the regression line), and a statement giving the Pearson correlation between the normalized intensity measurements in each sample.

### Other features

As a complement to the hierarchical clustering analysis, which may use either Euclidean distance or (1 - Pearson correlation) as the distance metric, PIIKA 2 also outputs files containing the Euclidean distance and Pearson correlation between each pair of samples, as well as each pair of subtracted treatment-control combinations. It may also be of interest to consider the distance between samples or treatment-control combinations by including in the calculation only peptides that are differentially phosphorylated. PIIKA 2 outputs files containing these data as well, with a peptide being considered differentially phosphorylated for a given pair of treatments or treatment-control combinations if the P-value according to the paired t-test is less than the user-specified threshold.

While PIIKA 2 contains many features related to the analysis and visualization of kinome microarray data, some users may want to perform analyses not available in PIIKA 2 or use their own visualization software. To facilitate this, PIIKA 2 outputs a file for each stage in the analysis pipeline containing the processed data at that stage. Specifically, a file is generated containing the data after background subtraction; after applying the vsn transformation; after rearranging the matrix; after averaging the technical and biological replicates; and after performing biological subtraction (if applicable). These files can easily be used as input to external programs.

### PIIKA 2 availability

PIIKA 2 is available both as a web server and as a stand-alone program that the user can run on his or her own computer. Each version has the same functionality, and can be accessed or downloaded via the SAskatchewan PHosphorylation Internet REsource (SAPHIRE) website at http://saphire.usask.ca.

The web-based version of PIIKA 2 is ideal for users who have limited experience with command line-based tools. To use the web-based version of PIIKA 2, the user must upload one or more input files, and enter the value of several parameters (number of intra-array replicates, number of peptides on the array, and so on). Detailed instructions for formatting the input files and choosing parameters are available on the PIIKA 2 webpage. The user must also enter his or her e-mail address; once the job has finished running, the user will receive an e-mail containing a link where the results can be downloaded. A full guide to the output of PIIKA 2 is available in File S1; a continuously updated version of the output guide is available via the SAPHIRE website, and is also included along with the other results files that the user downloads once their job is complete.

Commercial providers of kinome microarrays usually offer custom-designed arrays, where the client chooses the number of unique peptides to include on the array, the number of intra-array technical replicates per unique peptide, and the sequences of those peptides. Some providers also offer off-the-shelf arrays, for which the above attributes are predefined. To ease the submission process for those using the latter type, the PIIKA 2 website contains a drop-down menu where the user can select a particular off-the-shelf array. Once selected, the fields for certain parameters (the number of unique peptides on the array and the number of technical replicates per unique peptide) will be automatically filled in with the appropriate values. To identify off-the-shelf kinome arrays, we searched the websites of major providers of peptide arrays, including JPT Peptide Technologies (http://www.jpt.com), Pepscan (http://www.pepscan.com), Arrayit (http://www.arrayit.com), and PEPperPRINT (http://www.pepperprint.com).

The stand-alone version of PIIKA 2 is suitable for users familiar with command line-based tools, and requires that the R programming language [13] be installed, as well as several R packages. A full guide to installing and running the stand-alone version of PIIKA 2 is included in the download.

## Results

### Cluster analysis

**Figure 1:**
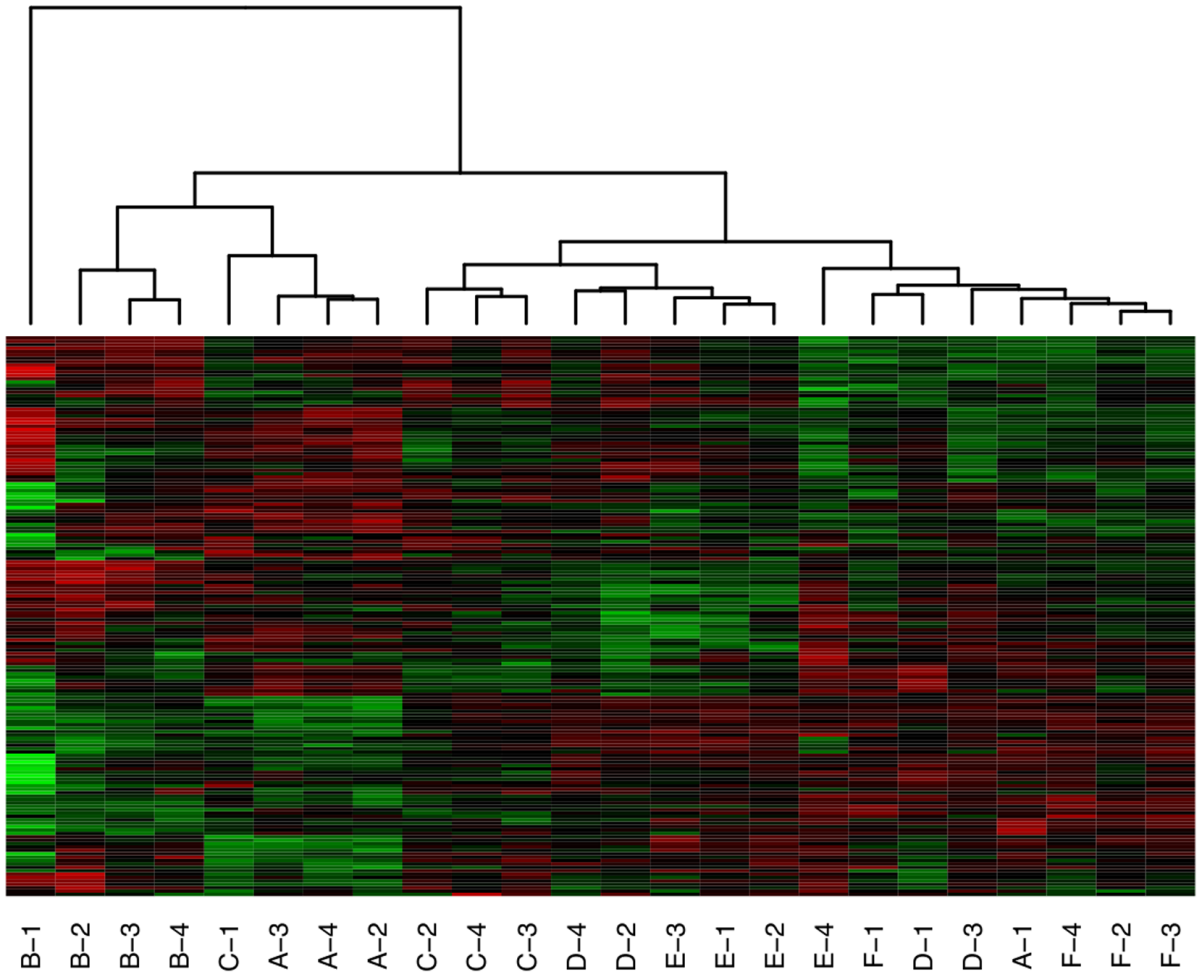
Heatmap and hierarchical clustering of kinome microarray profiles from the example experiment. Samples were taken at four time points from six different subjects, here labeled A-F. The number of the sample from the same subject represents the time point at which the sample was taken; for example, sample C-3 was taken from subject C at time point 3. The distance metric used for clustering was (1 - Pearson correlation), while the linkage method used was average linkage.

**Random tree analysis: statistical significance of the clustering of** ***a priori*** **groups.** To demonstrate the algorithm described in Methods, we use the aforementioned experimental data consisting of one sample taken at 4 time points from 6 subjects. The kinome array data were processed using the usual PIIKA pipeline (background subtraction followed by normalization and transformation using *vsn* [28]). Peptides that were consistently phosphorylated across the technical replicates according to a 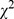-test for all 24 arrays (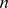  =  165) were then subjected to hierarchical clustering using (1 - Pearson correlation) as the distance metric and average linkage as the linkage method. The resulting heatmap is shown in Figure 1, with the sample (column) dendrogram showing that samples from the same subject tended to cluster together quite well, although not perfectly. The question is, do samples from the same subject cluster together better than would be expected by chance?

**Figure 2:**
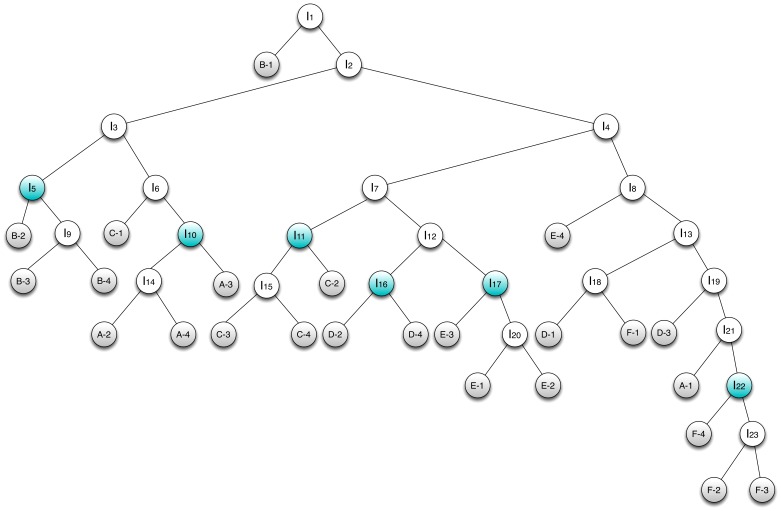
Binary tree representation of the dendrogram shown in Figure 1. Leaf nodes are shaded in grey and are labeled according to the subject and time point as in Figure 1. Internal nodes are labeled 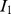 through 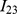, and those internal nodes 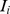 for which 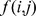 is maximized for some group 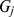 (where 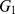 corresponds to subject A, 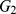 corresponds to subject B, and so on; see also Equation 1) are shaded in blue.

In our technique for ascertaining the statistical significance of the clustering of predefined groups, a hierarchical clustering is represented as a binary tree. As an example, the binary tree corresponding to the clustering shown in Figure 1 is shown in Figure 2. In applying Equation 1 to this tree, let subject A be 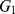, subject B be 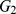, and so on. Then 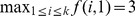, where 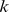 is the number of internal nodes. This expression is maximized when 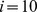, because internal node 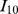 contains no leaves as descendants that correspond to any group other than 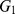 (subject A), and has three leaves as descendants that do correspond to 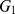 (the most of any internal node that satisfies the above condition). Similarly, 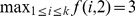, 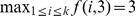, 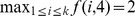, 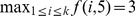, and 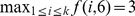. The sum of these is 17, and so 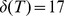 and 
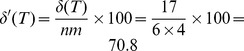
.

**Figure 3:**
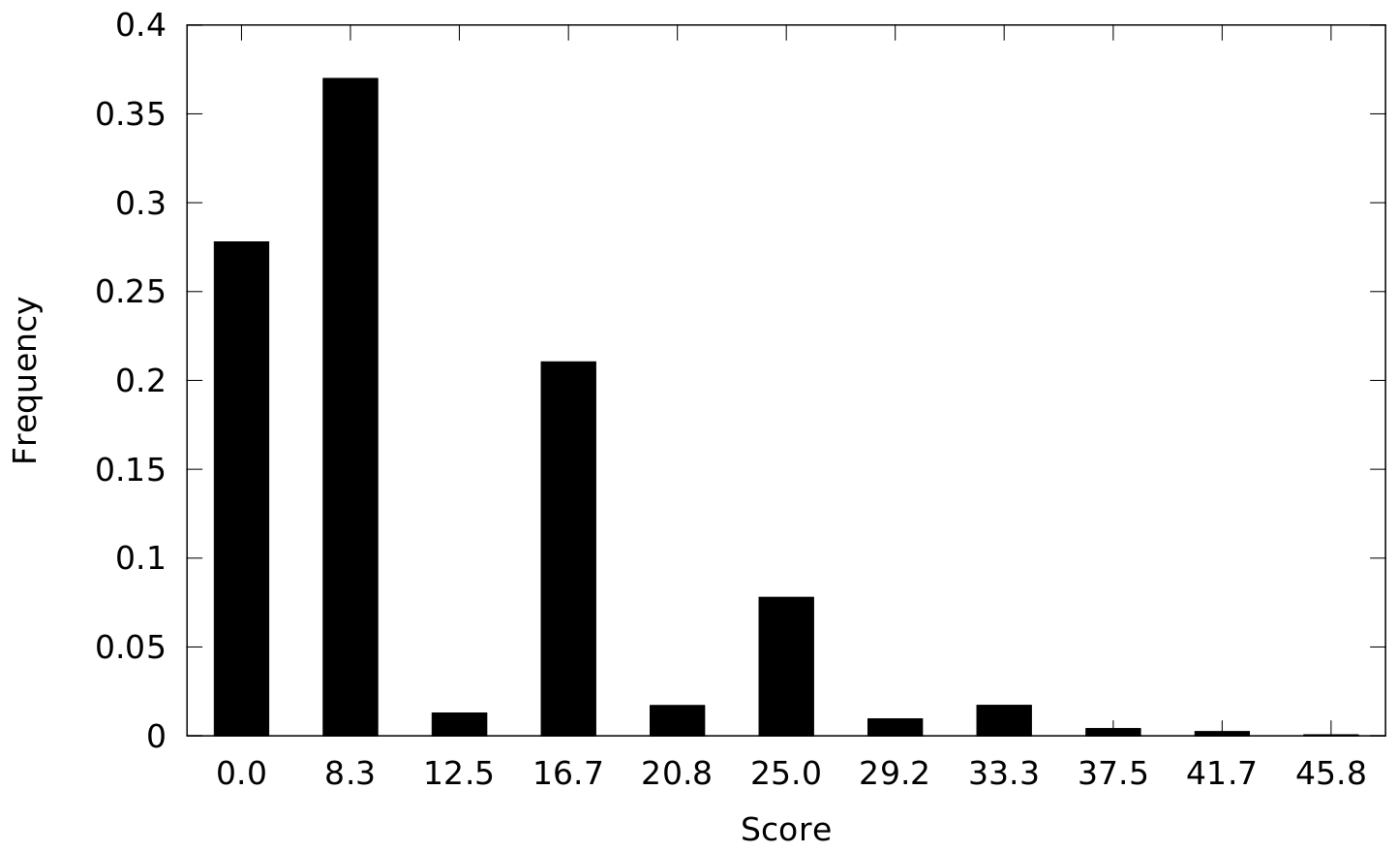
Empirical distribution of random tree scores. Ten thousand random matrices 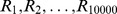 were created from the matrix used to create the sample dendrogram in Figure 1 by randomly rearranging the peptide intensity values within each sample. For each score 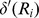 that was given to at least one random tree, the frequency of that score is indicated.

To generate the distribution of scores that would result by random chance, 10,000 random trees were generated by randomly rearranging the normalized intensity values for each peptide within a given array (column). The value of 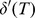 was calculated for each of these random trees, and the distribution of these data is shown in Figure 3. The lowest score given to a random tree was 0, while the greatest was 58.3. As such, none of the random trees had a score equal to or greater than the score for the actual tree, giving a P-value of less than 0.0001. This indicates that samples from the same subject do indeed cluster together better than would be expected by chance.

**Figure 4:**
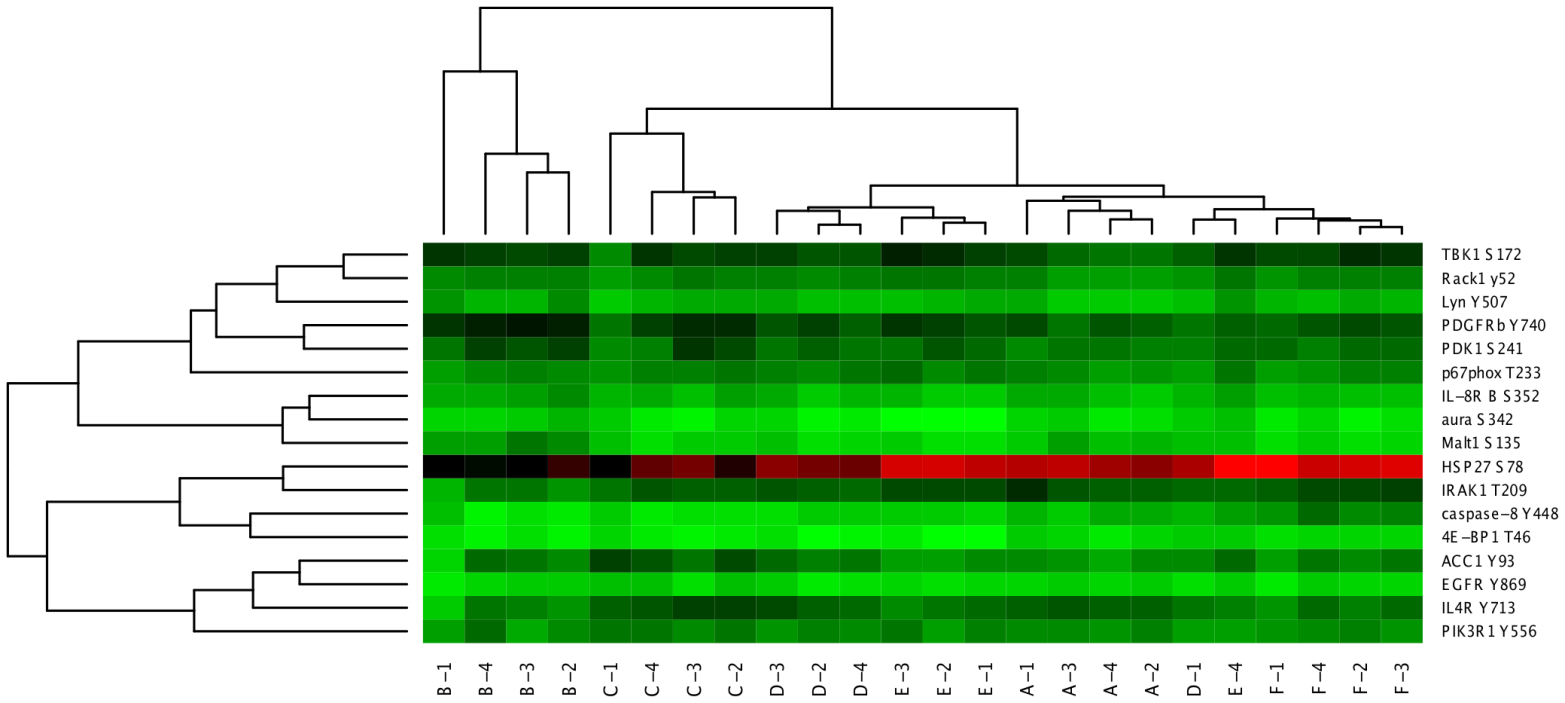
Heatmap and hierarchical clustering of kinome microarray profiles of samples from the example experiment using 17 peptides chosen according to a local search algorithm. The same distance metric and linkage method were used as in Figure 1. The sample names are the same as in Figure 1; the peptide names are also indicated on the right side of each row.

**Peptide subset analysis: identifying sets of peptides that support the clustering of** ***a priori*** **groups.** The local search procedure described in Methods was tested using the same sample data as described above. This procedure was used to identify sets of peptides that, when subjected to hierarchical clustering, resulted in a clustering with a value of 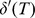 as close to 100 as possible—that is, a clustering where the arrays corresponding to a given subject cluster together, and cluster separately from arrays corresponding to other subjects. The greatest score 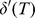 given to a dendrogram for some number of peptides 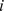 was 91.7, which was the case for 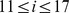. In other words, for each 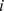 between 11 and 17 inclusive, a dendrogram could be created with 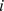 peptides that had a score of 91.7. The dendrogram corresponding to 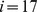 is shown in Figure 4. Figure 4 shows that, as its score suggests, the clustering with these 17 peptides is almost completely concordant with the “ideal” clustering by subject. Specifically, subjects A, B, C, and F all clustered together perfectly, while three out of the four samples from each of subjects D and E clustered together. As such, these 17 peptides were consistently phosphorylated within the same subject, but differentially phosphorylated between subjects.

**Figure 5:**
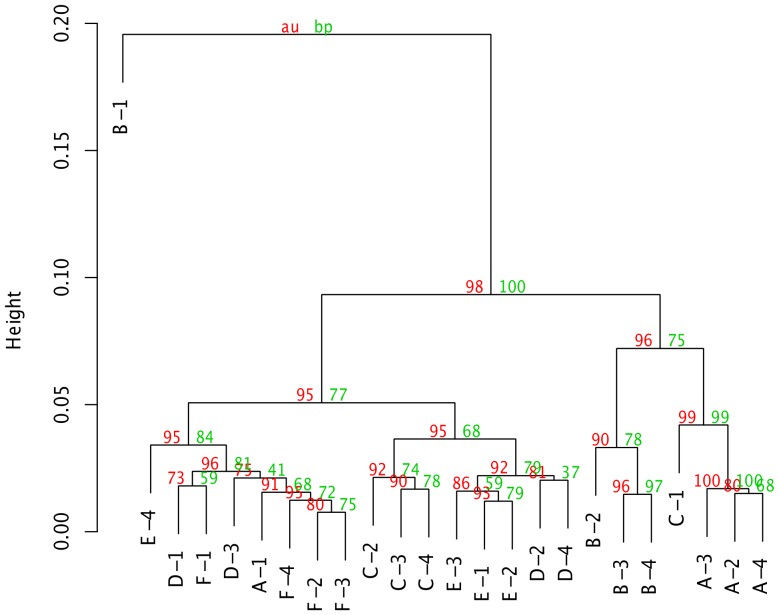
Example of a dendrogram with bootstrap values using PIIKA 2. The clustering of the samples is the same as in Figure 1. The red numbers represent the approximately unbiased (AU) P-values as determined using the method of Shimodaira [17], [18], while the green numbers represent the standard bootstrap P-value [16]. All calculations and the drawing of the figure were performed using the R package pvclust [19].

**Bootstrap analysis of hierarchical clustering.** One caveat with hierarchical clustering is that clusters are always produced, even in the extreme case where there is no relationship among any of the samples; as such, dendrograms containing bootstrap values represent valuable tools for assessing the strength and significance of the clusters produced. PIIKA 2 uses the R package pvclust [19] to generate dendrograms with bootstrap P-values on each node. These P-values are actually displayed as confidence values on the plot; for instance, a value of 99 means that the null hypothesis (“the cluster is not real”) can be rejected at a significance level of 0.01. An example of such a dendrogram, which was created using the same data and clustering methodology as the sample (column) dendrogram in Figure 1, is shown in Figure 5. For some of the subjects, the samples from the second, third, and fourth time points clustered together, while the sample from the first time point was an outlier (e.g. subject A). Figure 5 shows that, for some subjects, we could be very confident in the clustering of the latter three samples. For example, the cluster containing samples from the second, third, and fourth time points for subject A had a confidence value of 100. Conversely, there was somewhat less confidence for subject F, with the cluster containing the same three time points having an approximately unbiased confidence value of 95 but a standard bootstrap value of just 72.

### Statistical analysis

**False positive and false negative probabilities.** As described in Methods, PIIKA 2 now outputs values for 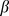 (the false positive rate) for each peptide for each treatment-control combination. These are present in the same files that contain the fold-change and t-test results. An example of such a file is given as Supplementary File S2.

**Positive and negative predictive values.** In addition to values for 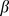, PIIKA 2 now also outputs positive and negative predictive values—the former being specific to a given treatment-control combination, and the latter being specific to each peptide within a given treatment-control combination. Like 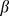, the negative predictive values are present in the file containing the fold-change and t-test results; see Supplementary File S2 for an example. Since the positive predictive value does not depend on the peptide, a separate file containing just the positive predictive value is generated for each treatment-control combination.

**Technical and biological reproducibility summaries.** As it is often of interest to determine and summarize the level of reproducibility of the intra-array technical replicates in a kinome microarray experiment, PIIKA 2 outputs a file containing the number of peptides for which the phosphorylation signal was determined to be consistent according to a 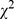-test for each array, as well as the range and average of these values. Supplementary File S3 contains an example of one of these files. If the experiment involves more than one biological replicate per treatment, then the level of reproducibility of these replicates may also be of interest; an example of such a summary can be found in Supplementary File S4.

### Data visualization

**Figure 6:**
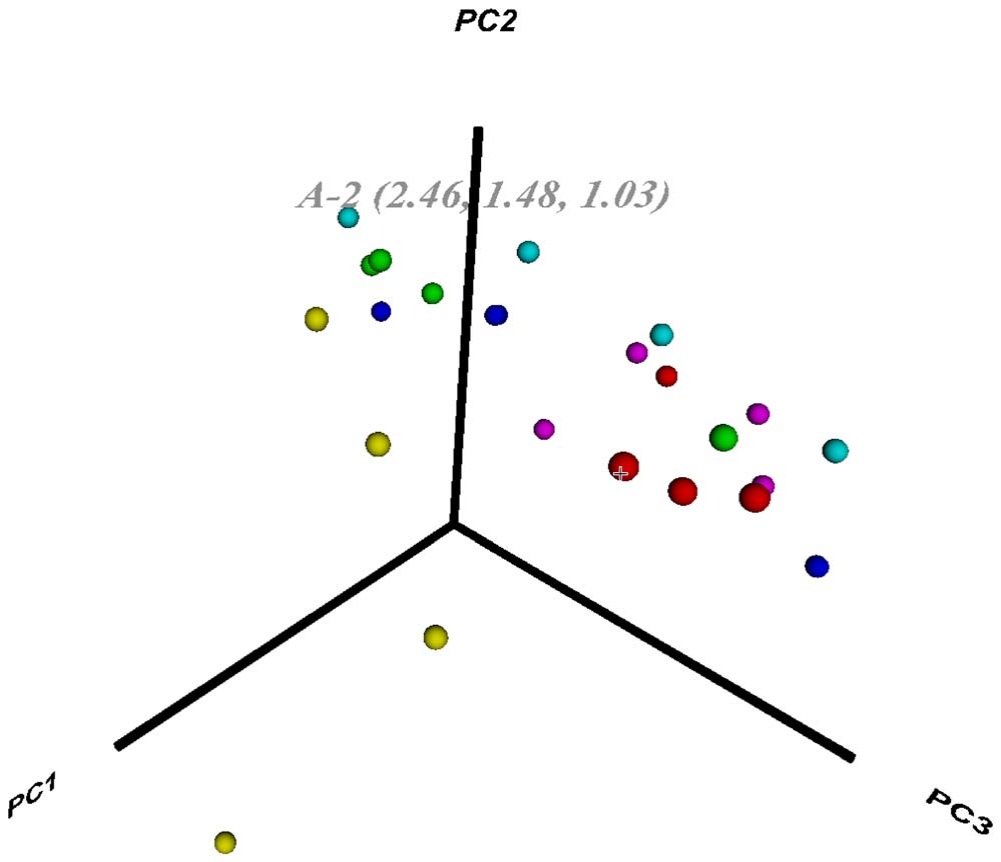
Example of a PCA plot generated in VRML format by PIIKA 2. In this experiment, samples were taken from subjects labeled A, B, C, D, E, and F. Samples corresponding to subject A are in red, subject B are in yellow, and so on. The label near the top of the figure is the result of hovering the mouse over the leftmost red circle, and shows that the first, second, and third principal components for this sample had the values 2.46, 1.48, and 1.03, respectively. This image is an example of the visualization given using the VRML viewer Instant Player ( http://www.instantreality.org ).

**PCA visualization using Virtual Reality Modeling Language.** A (static) picture of a VRML plot generated by PIIKA 2, as rendered by the visualization software Instant Player, is shown in Figure 6, and the corresponding VRML file is available as Supplementary File S5. The user has the option of assigning colours to each point in order to categorize them by treatment group, subject, etc. The user can also hover their mouse pointer over a given point to reveal the label corresponding to that point, as well as its coordinates (a three-tuple representing the values corresponding to the first, second, and third principal components, respectively). Collectively, these features allow users to more easily identify patterns in their data.

**Figure 7:**
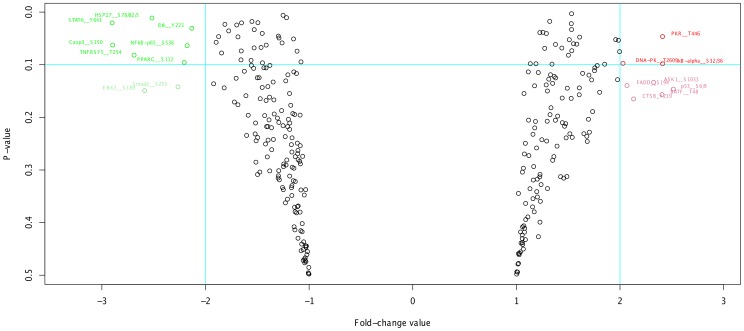
Example of a volcano plot generated using PIIKA 2. Points for which FC 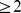 and P-value 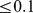 are coloured red, while those with FC 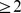 but P-value 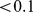 are pale red; Similarly, points with FC 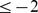 and P-value 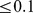 are green, while those with FC 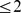 but P-value 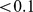 are pale green. All other points are coloured black. The horizontal and vertical blue lines represent the P-value and FC cutoffs, respectively. All coloured points are accompanied by labels showing to which peptide the point corresponds.

**Volcano plots.** For a given treatment-control combination, a volcano plot allows the user to easily identify peptides that both have a large FC value and have a significant P-value according to a t-test. An example of a volcano plot generated by PIIKA 2 is given in Figure 7. Each point has a specific colour depending on its FC value and P-value (see figure legend). In addition, all points having 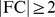 are labeled with their respective peptide names, allowing the user to easily identify peptides of interest.

**Figure 8:**
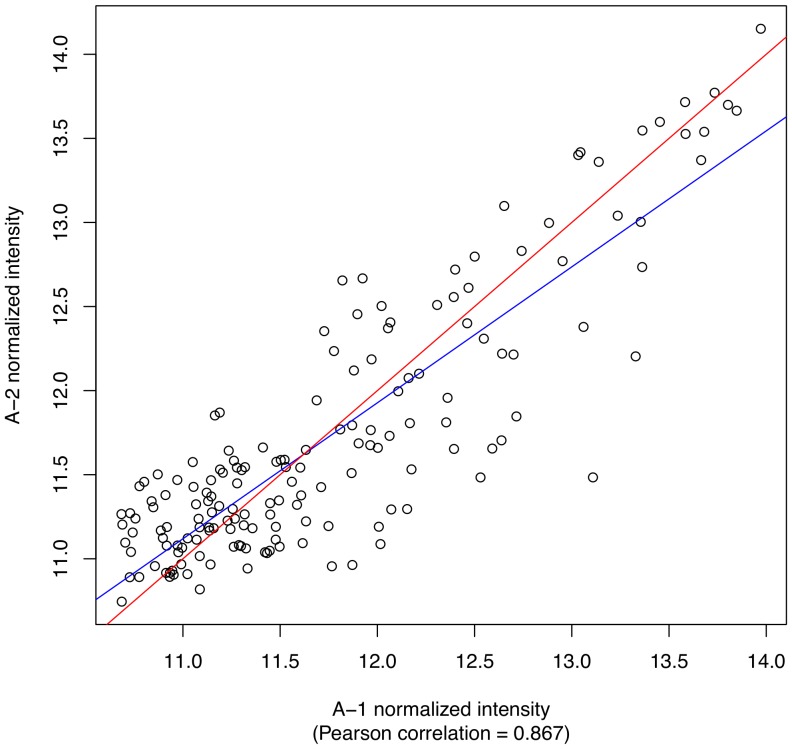
Example of a sample-sample scatterplot generated using PIIKA 2. Each point represents a peptide, and the 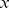 and 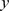 values of that point represent the normalized intensity values for that peptide for the first sample (A-1) and the second sample (A-2), respectively. The blue line represents the best fit using least squares, whereas the red line simply shows the diagonal (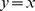). The Pearson correlation between the two samples is also indicated.

**Scatterplots between pairs of samples.** Figure 8 shows a sample scatterplot produced by PIIKA 2. The red and blue lines represent the diagonal (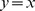) and the least squares regression line, respectively. The Pearson correlation coefficient is also shown below the 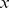-axis label.

### PIIKA 2 availability

**Figure 9:**
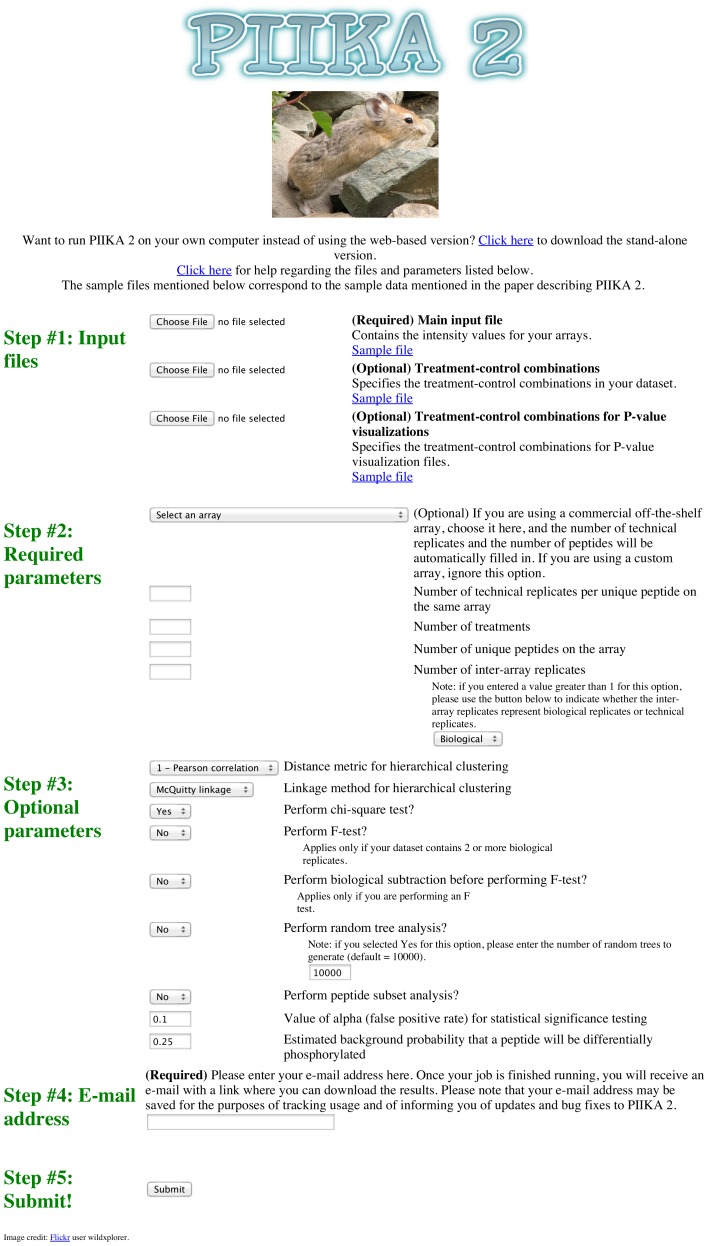
Screenshot of the user interface of the PIIKA 2 web server.

PIIKA 2 is available as a web server and as a stand-alone version, both of which can be accessed via http://saphire.usask.ca. Figure 9 contains a screenshot of the web server. As described in Methods, the web interface includes an option for the user to select an off-the-shelf kinome array purchased from a commercial provider, which allows the fields for certain parameters to be filled in automatically. Of the commercial providers mentioned in Methods, only JPT and Pepscan appeared to offer off-the-shelf kinome arrays, with JPT offering one array appropriate for use with PIIKA 2 and Pepscan offering three. Details on these arrays are given in Table 1. This feature will be expanded as more off-the-shelf commercial arrays become available.

**Table 1:** Off-the-shelf kinome microarrays that the PIIKA 2 web interface allows the user to select.

Company	Array name	Product code	# technical replicates	# peptides
JPT	Annotated Phosphosites-Kinase	KIN-MA-PhK	9	720
Pepscan	PepChip Kinomics Array	PCKINOM01	3	1024
Pepscan	PepChip Kinase Array	PCKF00020	2	1184
Pepscan	Kinase Evaluation Slide	PCKT00010	2	192

## Discussion and Conclusion

Many cellular processes can be regulated independently of changes in transcription or translation through post-translational modifications, the most important of which is kinase-mediated protein phosphorylation. Protein kinases play critical roles in regulating complex systems, underlie various pathologies, and represent high-priority drug targets; as such, there is considerable interest in defining and characterizing their biological roles. Kinome analysis offers three key advantages over traditional profiling of gene and/or protein expression: 1) individual kinase activities are often reliable indicators of phenotypic changes, 2) kinase profiling offers insight into cellular responses at the level of signaling networks, and 3) as kinases are highly “druggable”, increased understanding of their biological roles could aid therapeutic design and development.

The growing interest in kinases in both basic and translational research has driven efforts to develop technologies that facilitate the characterization of phosphorylation-mediated signal transduction. Peptide arrays are a relatively inexpensive technology that can be applied to study phosphorylation-mediated cellular signaling in a high-throughput manner. We and other groups have previously demonstrated the utility of kinome arrays for addressing a wide range of biological problems (e.g. [5]–[9], [15], [29], [30]). Given the substantial volume of data generated by kinome arrays, the ability to employ them effectively requires the existence of appropriate analysis methods. In this paper, we have described PIIKA 2, which is a powerful suite of tools for analyzing kinome microarray data. The new analysis tools have significant breadth, covering cluster analysis, statistical analysis, and data visualization. Further, we have provided an online submission platform that allows researchers to easily use PIIKA 2 for their own kinome investigations.

In this paper, the new features in PIIKA 2 were illustrated using a dataset derived from the application of kinome microarrays to real biological samples. However, few details about these samples were given, as this paper focuses on illustrating the capabilities of PIIKA 2, rather than reporting biological conclusions stemming from the analysis of this dataset. However, it should be emphasized that the value of PIIKA 2 lies primarily in its ability to help provide insight into biological systems. A concrete example of this is a recent study by our group that examined the kinome profiles of calf intestinal segments that were either infected or not infected with the bacterium *Mycobacterium avium* subsp. *paratuberculosis* [11]. In this study, PIIKA 2 was used to show that a given calf's kinome responses clustered into one of two groups, and the specific group to which a given calf belonged correlated with whether the animal exhibited primarily an antibody immune response or primarily a cell-mediated immune response.

As with any software package, future work will relate to the improvement or expansion of existing features, as well as the addition of new ones. Several of the additions and improvements to PIIKA 2 were inspired by, or have been useful for, our own research involving the application of kinome microarrays to biological problems. However, some of the questions other researchers wish to address may be different from our own. As such, we are interested in hearing from users of PIIKA 2 regarding ideas for additional features, as well as ways to improve the software in general.

## Supporting Information

File S1 **A guide to the output of PIIKA 2, listing all of the files produced by PIIKA 2, how they are organized, and what information is contained in each file.**
(PDF)

File S2
**A sample file containing results of a statistical comparison (fold-change values, P-values resulting from a paired t-test, values of** 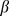 **, etc.) between a pair of samples from the example experiment.**
(TXT)

File S3 **A sample file containing a summary of the technical reproducibility of the arrays in the example experiment.**
(TXT)

File S4 **A sample file containing a summary of the reproducibility of the biological replicates in the example experiment.**
(TXT)

File S5 **A file in VRML format containing a 3D scatterplot of the first three principal components resulting from principal component analysis.** This file can be viewed using any VRML viewer, such as Instant Player (http://www.instantreality.org).
(VRML)
