# Correction: PIIKA 2: An Expanded, Web-Based Platform for Analysis of Kinome Microarray Data

**DOI:** 10.1371/annotation/8053077a-215f-4fa9-88ce-3c2be2795d12

**Published:** 2014-01-02

**Authors:** Brett Trost, Jason Kindrachuk, Pekka Määttänen, Scott Napper, Anthony Kusalik

There was an error in the first author's affiliation. Dr. Brett Trost is affiliated with: Department of Computer Science, University of Saskatchewan, Saskatoon, Saskatchewan, Canada.

