# Supplementary material for: PIIKA 2: An Expanded, Web-Based Platform for Analysis of Kinome Microarray Data
Source: PLoS One. 2013 Nov 29;8(11):e80837. doi: 10.1371/journal.pone.0080837 (PMC3843739; doi:10.1371/journal.pone.0080837)
Supplement: File S1 — A guide to the output of PIIKA 2, listing all of the files produced by PIIKA 2, how they are organized, and what information is contained in each file. (PDF) [file pone.0080837.s001.pdf]

PIIKA 2 output guide

This document describes the output produced by PIIKA 2. The .zip file your downloaded contains several directories. Descriptions of these directories (represented by the headings below), as well as the files contained in these directories, are given below. Depending on the options you selected when you ran PIIKA, some of the directories listed below may be absent.

Several of the directories contain analyses performed after "biological subtraction", which we abbreviate in filenames as "biosub". Biological subtraction means that for each treatment-control combination specified by the user, the normalized intensity value for the control is subtracted from the normalized intensity value for the treatment for each peptide. The particular analysis being described is then performed on these subtracted values. Analyses involving biological subtraction are performed only if the user uploads a file specifying the treatment-control combinations present in the data.

Two files are contained within the top-level directory, rather than being contained within some subdirectory. These are:

- `parameters.txt`—Contains the value of the parameters used to PIIKA 2.
- `PIIKA2_output_guide.pdf`—This document.

PCA

Contains files related to Principal Component Analysis (PCA) of the various treatments.

- `PCA.txt`—A table in tab-delimited text format containing the values for the first three principal components for each treatment.
- `PCA.vrml`—Contains a 3D visualization of the PCA for each treatment in Virtual Reality Modeling Language (VRML) format. To view this file, you can use a VRML viewer such as Instant Player (`http://www.instantreality.org`).
- `PCA.vrml.legend.pdf`—Gives the colour by which each treatment is represented in `PCA.vrml`. This file will only be meaningful if your samples are named such that they indicate defined groups. For more information, see http://saphire.usask.ca/saphire/piika/piika2_input_guide.html.
- `PCA_PC1_PC2.pdf`—A two-dimensional scatterplot depicting the first two principal components, with the coordinates coming from `PCA.txt`.
- `PCA_PC2_PC3.pdf`—A two-dimensional scatterplot depicting the second and third principal components, with the coordinates coming from `PCA.txt`.
- `PCA_PC1_PC2_PC3.pdf`—A three-dimensional scatterplot depicting the first three principal components, with the coordinates coming from `PCA.txt`.

PCA_biosub

This directory will be present only if a file is uploaded for the "treatment-control combinations" field.

Contains files related to PCA of the various treatment-control combinations.

- `PCA_biosub.txt`—A table in tab-delimited text format containing the values for the first three principal components for each treatment-control combination.
- `PCA_biosub.vrml`—Contains a 3D visualization of the PCA for each treatment-control combination in Virtual Reality Modeling Language (VRML) format. To view this file, you can use a VRML viewer such as Instant Player (`http://www.instantreality.org`).
- `PCA_PC1_PC2.pdf`—A two-dimensional scatterplot depicting the first two principal components, with the coordinates coming from `PCA_biosub.txt`.
- `PCA_PC2_PC3.pdf`—A two-dimensional scatterplot depicting the second and third principal components, with the coordinates coming from `PCA_biosub.txt`.
- `PCA_PC1_PC2_PC3.pdf`—A three-dimensional scatterplot depicting the first three principal components, with the coordinates coming from `PCA_biosub.txt`.

biological_reproducibility

This directory will be present only if the "Perform F test?" option is set to "Yes".

Contains files relating to the biological reproducibility of the array data (i.e. the consistency of the phosphorylation signal for each peptide in the different animals (biological replicates) for which the experiment was performed).

- `F_test_consistent_peptides.txt`—For each peptide, its value will be "TRUE" if that peptide is consistent according to the F test for all treatments, and "FALSE" otherwise.
- `F_test_pvalues.txt`—Contains the P-value according to the F test for each peptide for each treatment.
- `biological_reproducibility_summary.txt`—Gives the number of peptides that were biologically consistent according to the F test for each treatment, as well as the range of values and average of these values.

distances

Contains files giving numeric representations of the similarity of pairs of samples.

- `distances_euclidean.txt`—For each pair of samples, contains the Euclidean distance between that pair. Let n represent the number of peptides. Then the Euclidean distance is calculated as $\sqrt{\sum_{i=1}^{n}(x_i - y_i)^2}$, where x_i is the averaged (among all technical and biological replicates) intensity level for peptide i for the first sample, and y_i is the corresponding value for the second sample.
- `distances_pearson.txt`—For each pair of samples, contains the value (1 - Pearson correlation) for that pair. This is calculated using the `cor` function in R with `method = "pearson"`.

distances_biosub

This directory will be present only if a file is uploaded for the "treatment-control combinations" field.

Contains files giving numeric representations of the similarity of pairs of treatment-control combinations.

- `distances_biosub_euclidean.txt`—For each pair of treatment-control combinations, contains the Euclidean distance between that pair. Let n represent the number of peptides. Then the Euclidean distance is calculated as $\sqrt{\sum_{i=1}^{n}(x_i - y_i)^2}$, where x_i is the averaged (among all technical and biological replicates) intensity level for peptide i for the first treatment-control combination, and y_i is the corresponding value for the second treatment-control combination.

- `distances_biosub_pearson.txt`—For each pair of treatment-control combinations, contains the value (1 - Pearson correlation) for that pair. This is calculated using the `cor` function in R with `method = "pearson"`.

distances_significant

Contains files giving numeric representations of the similarity of pairs of samples, but taking into account only the peptides that have a statistically significant difference in phosphorylation for that pair.

- `distances_euclidean.txt`—For each pair of samples, contains the Euclidean distance between that pair, taking into account only the peptides for which the P-value from the paired t-test is less than the user-specified threshold. So that different pairs of samples can be compared, this value is then normalized by the number of significant peptides for that pair. Let n represent the number of peptides for which the paired t-test gives a P-value less than the specified threshold. Then the normalized Euclidean distance is calculated as $\frac{1}{n}\sqrt{\sum_{i=1}^{n}(x_i - y_i)^2}$, where x_i is the averaged (among all technical and biological replicates) intensity level for peptide i for the first sample, and y_i is the corresponding value for the second sample.

- `distances_pearson.txt`—For each pair of samples, contains the value (1 - Pearson correlation) for that pair. This is calculated using the `cor` function in R with `method = "pearson"`. As with the Euclidean distance, only peptides for which the P-value from the paired t-test is less than the user-specified threshold are used in the calculation, and the resulting value is divided by the number of significant peptides so that different pairs of samples can be compared on the same scale.

distances_biosub_significant

This directory will be present only if a file is uploaded for the "treatment-control combinations" field.

Contains files giving numeric representations of the similarity of pairs of treatment-control combinations, but taking into account only the peptides that have a statistically significant difference in phosphorylation (after biological subtraction) for that pair.

- `distances_biosub_significant_euclidean.txt`—For each pair of treatment-control combinations, contains the Euclidean distance between that pair, taking into account only the peptides for which the P-value from the paired t-test is less than the user-specified threshold. So that different pairs of treatment-control combinations can be compared, this value is then normalized by the number of significant peptides for that pair. Let n represent the number of peptides for which the paired t-test gives a P-value less than the specified threshold. Then the normalized Euclidean distance is calculated as $\frac{1}{n}\sqrt{\sum_{i=1}^{n}(x_i - y_i)^2}$, where x_i is the averaged (among all technical and biological replicates) intensity level for peptide i for the first treatment-control combination, and y_i is the corresponding value for the second treatment-control combination.

- `distances_biosub_significant_pearson.txt`—For each pair of treatment-control combinations, contains the value (1 - Pearson correlation) for that pair. This is calculated using the `cor` function in R with `method = "pearson"`. As with the Euclidean distance, only peptides for which the P-value from the paired t-test is less than the user-specified threshold are used in the calculation, and the resulting value is divided by the number of significant peptides so that different pairs of treatment-control combinations can be compared on the same scale.

hierarchical_clustering

Contains files relating to the hierarchical clustering of the samples and peptides. These files are constructed using the distance metric and linkage method chosen by the user, with the defaults being (1 - Pearson correlation) and McQuitty linkage, respectively.

- `bootstrap_dendrogram.pdf`—Contains a dendrogram depicting the hierarchical clustering of the samples, with bootstrap values as calculated using the R package `pvclust`.

- `heatmap.pdf`—Contains a heatmap wherein the columns represent samples, the rows represent peptides, and the color of the cells represent degree of up-phosphorylation (red) or down-phosphorylation (green). The top dendrogram represents the clustering of the samples, and the left dendrogram represents the clustering of the peptides.

- `heatmap.sample_dendrogram.txt`—A text-based version of the sample dendrogram depicted in the file `heatmap_euclidean_average.pdf`.

- `heatmap.peptide_dendrogram.txt`—A text-based version of the peptide dendrogram depicted in the file `heatmap_euclidean_average.pdf`.

hierarchical_clustering_biosub

This directory will be present only if a file is uploaded for the "treatment-control combinations" field.

Contains files relating to the hierarchical clustering of the treatment-control combinations and peptides. These files are constructed using the distance metric and linkage method chosen by the user, with the defaults being (1 - Pearson correlation) and McQuitty linkage, respectively.

- `bootstrap_dendrogram_biosub.pdf`—Contains a dendrogram depicting the hierarchical clustering of the treatment-control combinations, with bootstrap values as calculated using the R package `pvclust`.

- `heatmap_biosub.pdf`—Contains a heatmap wherein the columns represent treatment-control combinations, the rows represent peptides, and the color of the cells represent degree of up-phosphorylation (red) or down-phosphorylation (green) after biological subtraction. The top dendrogram represents the clustering of the treatment-control combinations, and the left dendrogram represents the clustering of the peptides.

- `heatmap_biosub.sample_dendrogram.txt`—A text-based version of the sample dendrogram depicted in the file `heatmap_biosub.pdf`.

- `heatmap_biosub.peptide_dendrogram.txt`—A text-based version of the peptide dendrogram depicted in the file `heatmap_biosub.pdf`.

intermediate_results

Contains files giving various intermediate results as the data are processed by PIIKA 2.

- `step1_raw_data.txt`—Contains the raw intensity data for each peptide for each array (foreground and background values), identical to the file uploaded by the user in the "Main input file" field.
- `step2_background_corrected.txt`—Contains the intensity value for each peptide for each array after subtracting the background from the foreground.
- `step3_vsn.txt`—Contains the normalized intensity value (normalization using the *vsn* method) for each peptide for each array.
- `step4_rearranged.txt`—Contains the same data as in `step3_vsn.txt`, except the matrix has been rearranged such that all of the intensity values corresponding to a particular peptide are in the same row.
- `step5_averages.txt`—Contains the average normalized intensity value for each treatment for each peptide.
- `step5_averages.consistent.txt`—Contains the average normalized intensity value for each treatment for each peptide that was consistent for all arrays according to the chi-square test (if applicable), and for all animals according to the F-test (if applicable).
- `step6_biosub_averages.txt`—For each treatment-control combination, this matrix contains the subtracted value (average value for treatment minus average value for control) for each peptide. *This file will be present only if a file is uploaded for the "treatment-control combinations" field.*
- `step6_biosub_averages.consistent.txt`—For each treatment-control combination, this matrix contains the subtracted value (average value for treatment minus average value for control) for each peptide that was consistent for all arrays according to the chi-square test (if applicable), and for all animals according to the F-test (if applicable). *This file will be present only if a file is uploaded for the "treatment-control combinations" field.*

scatterplots

For each pair of samples, contains a scatterplot depicting the averaged normalized intensity for each peptide for each sample in that pair.

- `<sample1>_vrs_<sample2>.pdf`—A scatterplot depicting the relationship between the averaged normalized intensity values for sample 1 and the averaged normalized intensity values for sample 2.

scatterplots_biosub

For each pair of treatment-control combinations, contains a scatterplot depicting the averaged normalized intensity for each peptide for each treatment-control combination in that pair.

- `<treatment-control_combination1>_vrs_<treatment-control_combination2>.pdf`—A scatterplot depicting the relationship between the averaged normalized intensity values for the first treatment-control combination and the averaged normalized intensity values for the second treatment-control combination.

t-tests

Contains files relating to the statistical significance of differences in phosphorylation between each treatment and control.

- <sample1>_vrs_<sample2>.all.txt—A table in tab-delimited text format giving various statistical measures of the difference in phosphorylation of each peptide in sample 1 (treatment) versus sample 2 (control). The peptides are sorted in order of increasing P-value (where this P-value is the smaller of the P-value for up-phosphorylation or down-phosphorylation). The first row contains column headings, the meanings of which are described below.
  - ID—The name of the protein from which the peptide is derived.
  - Accession—The accession number of that protein.
  - FC—The fold-change value for the peptide in the treatment versus the control.
  - P up—The P-value for up-phosphorylation in the treatment compared to the control according to the paired t-test.
  - P down—The P-value for down-phosphorylation in the treatment compared to the control according to the paired t-test.
  - Beta up—The value of β for up-phosphorylation in the treatment compared to the control.
  - Beta down—The value of β for down-phosphorylation in the treatment compared to the control.
  - Negative predictive value up—The negative predictive value for up-phosphorylation in the treatment compared to the control.
  - Negative predictive value down—The negative predictive value for down-phosphorylation in the treatment compared to the control.
- <sample1>_vrs_<sample2>.all.unsorted.txt—The same as <sample1>_vrs_<sample2>.all.txt, except not sorted by P-value.
- <sample1>_vrs_<sample2>.consistent.txt—The same as <sample1>_vrs_<sample2>.all.txt, except lists only peptides that are consistently phosphorylated in both the treatment and the control (if the χ^2 test was done), and which were consistently phosphorylated among the biological replicates for both treatment and control (if the F test was done). *This file will be present only if one or both of the "Perform chi-square test?" or "Perform F test" options are set to "Yes".*
- <sample1>_vrs_<sample2>.significant.txt—The same as <sample1> _vrs_<sample2>.all.txt, except lists only peptides that have a P-value for either up-phosphorylation or down-phosphorylation less than the user-specified threshold.
- <sample1>_vrs_<sample2>.consistent_significant.txt—Contains only the peptides listed in both <sample1>_vrs_<sample2>.consistent.txt and <sample1>_vrs_<sample2>.significant.txt. *This file will be present only if one or both of the "Perform chi-square test?" or "Perform F test" options are set to "Yes".*
- <sample1>_vrs_<sample2>.volcano.pdf—A volcano plot, which is a scatterplot with fold-change values on the x-axis and P-values on the y-axis.
- <sample1>_vrs_<sample2>.consistent.volcano.pdf—The same as <sample1>_vrs_<sample2>.volcano.pdf, except only shows peptides listed in <sample1>_vrs_<sample2>.consistent.txt. *This file will be present only if one or both of the "Perform chi-square test?" or "Perform F test" options are set to "Yes".*
- <sample1>_vrs_<sample2>.not_significant.txt—Contains only the peptides not listed in <sample1> _vrs_<sample2>.significant.txt.

- <sample1>_vrs_<sample2>.positive_predictive_value.txt—Contains the positive predictive value for this treatment-control combination (which is the same for all peptides).

technical_reproducibility

This directory will be present only if the "Perform chi-square test?" option is set to "Yes".

Contains files relating to the technical reproducibility of the array data (i.e. the consistency of the phosphorylation signal for identical peptides replicated multiple times on the same array).

- chi_square_test_consistent_peptides.txt—For each peptide, its value will be "TRUE" if that peptide is consistent according to the χ^2 test for all arrays, and "FALSE" otherwise.
- chi_square_test_pvalues.txt—Contains the P-value according to the χ^2 test for each peptide for each array.
- technical_reproducibility_summary.txt—Gives the number of peptides on each array that were technically consistent according to the χ^2 test for each array, as well as the range of values and average of these values.

random_trees

This directory will be present only if the "Perform random tree analysis?" option is set to "Yes".

Contains files related to the random tree analysis described in the main paper, which seeks to answer the question, "Do the samples cluster together better than would be expected by chance?". These files are constructed using the distance metric and linkage method chosen by the user, with the defaults being (1 - Pearson correlation) and McQuitty linkage, respectively.

- heatmap_random_<n>.averages.txt—For the nth random dendrogram, contains the randomly-rearranged matrix used to generate that dendrogram.
- heatmap_random_<n>.pdf—For the nth random dendrogram, contains the heatmap depicting that dendrogram.
- heatmap_random_<n>.sample_dendrogram.txt—For the nth random dendrogram, contains a text-based version of that dendrogram.
- heatmap_random_tree_pvalue.txt—Contains the P-value, which indicates the likelihood that the clustering of the actual tree (the dendrogram found in the hierarchical_clustering directory) was better than would be expected by chance. The P-value is calculated as the proportion of random trees that got scores equal to or greater than the score for the actual tree.
- heatmap_random_tree_scores.txt—Lists the score associated with each random tree.

peptide_subset_analysis

This directory will be present only if the "Perform peptide subset analysis?" option is set to "Yes".

Contains files related to the peptide subset analysis described in the main paper, which seeks to answer the question, "What subsets of the peptides give perfect or near-perfect clustering of the samples?". These files are constructed using the distance metric and linkage method chosen by the user, with the defaults being (1 - Pearson correlation) and McQuitty linkage, respectively.

- `best_set_<n>.heatmap.pdf`—Contains a heatmap generated using the n peptides found to have the best tree score.
- `best_set_<n>.peptides.txt`—Contains the n peptides found to have the best tree score.
- `best_set_<n>.sample_dendrogram.txt`—Contains a text-based version of the sample dendrogram generated using the n peptides found to have the best tree score.
- `best_set_<n>.score.txt`— contains the best tree score when using n peptides.
